# Functional and Pharmacological Characterization of the Rare CFTR Mutation W361R

**DOI:** 10.3389/fphar.2020.00295

**Published:** 2020-03-17

**Authors:** Arnaud Billet, Ahmad Elbahnsi, Mathilde Jollivet-Souchet, Brice Hoffmann, Jean-Paul Mornon, Isabelle Callebaut, Frédéric Becq

**Affiliations:** ^1^Laboratoire Signalisation et Transports Ioniques Membranaires, Université de Poitiers, CNRS, Poitiers, France; ^2^Sorbonne Université, Muséum National d’Histoire Naturelle, UMR CNRS 7590, Institut de Minéralogie, de Physique des Matériaux et de Cosmochimie, IMPMC, Paris, France

**Keywords:** W361R-CFTR, missense CF mutations, class-2 CF mutations, small molecules modulators, cystic fibrosis transmembrane conductance regulator

## Abstract

Understanding the functional consequence of rare cystic fibrosis (CF) mutations is mandatory for the adoption of precision therapeutic approaches for CF. Here we studied the effect of the very rare CF mutation, W361R, on CFTR processing and function. We applied western blot, patch clamp and pharmacological modulators of CFTR to study the maturation and ion transport properties of pEGFP-WT and mutant CFTR constructs, W361R, F508del and L69H-CFTR, expressed in HEK293 cells. Structural analyses were also performed to study the molecular environment of the W361 residue. Western blot showed that W361R-CFTR was not efficiently processed to a mature band C, similar to F508del CFTR, but unlike F508del CFTR, it did exhibit significant transport activity at the cell surface in response to cAMP agonists. Importantly, W361R-CFTR also responded well to CFTR modulators: its maturation defect was efficiently corrected by VX-809 treatment and its channel activity further potentiated by VX-770. Based on these results, we postulate that W361R is a novel class-2 CF mutation that causes abnormal protein maturation which can be corrected by VX-809, and additionally potentiated by VX-770, two FDA-approved small molecules. At the structural level, W361 is located within a class-2 CF mutation hotspot that includes other mutations that induce variable disease severity. Analysis of the 3D structure of CFTR within a lipid environment indicated that W361, together with other mutations located in this hotspot, is at the edge of a groove which stably accommodates lipid acyl chains. We suggest this lipid environment impacts CFTR folding, maturation and response to CFTR modulators.

## Introduction

Cystic fibrosis (CF) is one of the most common, lethal, autosomal recessive disease and is caused by mutations in the gene encoding the anion channel cystic fibrosis transmembrane conductance regulator (CFTR). CFTR is a major actor in transepithelial fluid secretion and a defect in its expression, location or function results in severe pulmonary and digestive impairments (Rowe et al., 2005). More than 2000 CF mutations have been identified to date, and these have been subdivided into six classes based on the functional defect they cause on CFTR mRNA or protein (Kerem, 2006). The six classes are: (1) defective protein synthesis, (2) impaired protein maturation leading to protein degradation, (3) defective regulation of CFTR channel activity, (4) altered ionic selectivity and conductance, (5) lowered CFTR mRNA levels, and (6) decreased protein stability.

The most common CF mutation is a deletion of phenylalanine at position 508 (F508del), which is a class-2 mutation exhibiting inefficient maturation and reduced plasma membrane expression of the protein (Cheng et al., 1990; Denning et al., 1992). F508del-CFTR also exhibits a gating defect (Dalemans et al., 1991), and a reduced stability at the plasma membrane (Lukacs et al., 1993; Okiyoneda et al., 2010), typical defects of class-3 and class-6 mutations, respectively. This mutation is present in 70% of homozygous patients and 90% of heterozygous patients [CFTR2 database^1^ ]. Patients carrying class-2 CFTR mutations on both alleles generally show severe disease with strong digestive and pulmonary defects.

Here we studied a very rare CF mutation, W361R (NM_000492.3:c.1081T > A), a variant of CFTR described in only a small number of European patients; Spanish, French, Italian (Alonso et al., 2007), Greek (Kanavakis et al., 1995), and Swedish (Strandvik et al., 2001). This mutation was first detected in a French woman (Bienvenu et al., 1994), who had limited respiratory problems during childhood. She was only diagnosed with CF at the age of 37 years, and later died at the age of 44 because of pulmonary decompensation. She was pancreatic sufficient but developed late-onset severe lung disease. She carried a splicing mutation 297-3C > T in intron 2 on the other allele.

In order to gain a better understanding of the effect of CF mutations on CFTR function, and especially why some patients carrying some rare, or very rare, mutations only develop severe symptoms later in life, we examined the functional and cellular consequences of the rare missense mutation, W361R, and compared it to F508del-CFTR, and another class 2 CF mutation, L69H, which causes a severe CF phenotype with pancreatic insufficiency and congenital bilateral absence of the vas deferens (CBAVD) (Sharma et al., 2015).

## Materials and Methods

### Cell Culture

HEK293 cells were cultured at 37°C in 5% CO_2_ in Dulbecco’s modified Eagle’s medium + GlutaMAX (Life Technologies, United Kingdom) with 10% fetal calf serum (Lonza, Belgium), 1% penicillin/streptomycin (Sigma-Aldrich).

### Construction and Expression of CFTR Mutants

The expression vectors used include pEGFP-CFTR-WT and pEGFP-CFTR-F508 plasmids generously provided by K. H. Karlson (Dartmouth College, Hanover, NH) (Moyer et al., 1998). The W361R mutation was generated by site-directed mutagenesis as previously described (Billet et al., 2010) using the oligonucleotide primer (T to A) 5′-CCCTGGGCTGTACAAACAAGGTATGACTCTCTTGGAGC-3′. Cells were transiently transfected using cationic lipids (JetPEI DNA Transfection Reagent, Polyplus transfection, Illkirch, France) with 0.5 μg/mL (patch-clamp) or 1 μg/mL (western blot analysis) of plasmid.

### Western Blotting

After 48 h of transfection, HEK293 cells were lysed (lysis buffer: 10 mM Tris HCl, 1% Non-idet P-40, 0.5% sodium deoxycholate, 1 mM Pefabloc^®^ SC and the protease inhibitors cocktail cOmplete^TM^ [Roche, Germany); pH 7.5)]. 50 μg of protein were resolved on a 5–10% gradient SDS-PAGE, transferred to a nitrocellulose membrane, probed using the MAB3480 anti-CFTR antibody (a.a 1370–1380, clone M3A7) and the Na/K ATPase (1:1000; Millipore Corporation, United States), then incubated with a secondary peroxidase-conjugated antibody (1:5000; Amersham, GE Healthcare, United Kingdom) followed by chemiluminescence detection.

Maturation level was quantified by densitometry using ImageJ software (Wayne Rasband, National Institute of Health, United States) and normalized to the loading control, the Na/K ATPase protein.

### Patch Clamp Recording

Ionic currents were recorded using the whole-cell and the excised inside out configurations of the patch-clamp method.

Whole cell experiments were conducted as previously described (Billet et al., 2010). The external bath solution contained (in mM) 145 NaCl, 4 CsCl, 1 CaCl_2_, 1 MgCl_2_, 10 glucose, and 10 N-tris[Hydroxymethyl]methyl-2-aminoethanesulphonic acid (TES) (titrated with NaOH to pH 7.4). The intrapipette solution contained (in mM) 113 L-aspartic acid, 113 CsOH, 27 CsCl, 1 NaCl, 1 MgCl_2_, 1 EGTA, 10 TES, and 3 MgATP (titrated with CsOH to pH 7.2). In all experiments, CFTR channels were activated by elevating intracellular cAMP levels using 10 μM of the adenylate cyclase agonist forskolin (Fsk) and currents potentiated using 30 μM genistein (Gst) (Illek et al., 1995). Then, the thiazolidinone blocker CFTRinh-172 was used at 10 μM to selectively inhibit CFTR channels (Ma et al., 2002). All experiments were conducted at room temperature (20–25°C).

Microscopic CFTR currents were recorded from inside-out patches with the pipette potential held at + 50 mV (i.e., membrane potential, –50 mV) and inverted for purposes of illustration. The pipette (extracellular) solution contained (mM): 140 N-methyl-D-glucamine (NMDG), 140 aspartic acid, 5 CaCl_2_, 2 MgSO_4_ and 10 TES (titrated with Tris to pH 7.3). The bath (intracellular) solution contained (mM): 140 NMDG, 3 MgCl_2_, 1 CsEGTA, and 10 TES (titrated with HCl to pH 7.3). Currents were recorded at 500 Hz and filtered at 125 Hz using an Axopatch 200B amplifier and an analog/digital interface (Digidata 1440A) and analyzed with pCLAMP 10 software (all from Axon Instruments, Inc., Burlingame, CA, United States). CFTR channels were activated by adding 1 mM MgATP and 75 nM PKA to the bath solution. Experiments were conducted at room temperature (20–25°C).

### Chemicals

Forskolin (Fsk), genistein (Gst), and CFTR-_*inh*_172 were purchased from Sigma-Aldrich. VX-770 and VX-809 were purchased from Euromedex (Souffelweyersheim, France). Stock solutions of Fsk (10 mM), Gst (30 mM), CFTRinh172 (10 mM), VX-770 (1 mM), and VX-809 (10 mM) were prepared in dimethyl sulfoxide (DMSO). PKA (final concentration, 75 nM – 30 U) was from Promega (Madison, WI, United States); MgATP (1 mM) was from Sigma-Aldrich.

### Molecular Dynamics Simulations

Equilibrium molecular dynamics (MD) simulations were carried out with the NAMD 2.9 program (Phillips et al., 2005), using the CHARMM36 force field (Hart et al., 2012) as detailed in a previous article (Hoffmann et al., 2018). Cryo-EM- (pdb:6MSM) or model-derived 3D structures of human CFTR were embedded in a POPC lipid bilayer and immersed in an orthorhombic box filled with TIP3P water using the charmm-gui platform (Jo et al., 2008; Wu et al., 2014; Lee et al., 2016). Electrical neutrality was achieved by adding Na^+^ and Cl^–^ ions to reach a final concentration of 150 mM. After equilibration steps, the production phases were carried out in the NPT ensemble at a temperature of 310 K and a pressure of 1 bar, using periodic boundary conditions and Particle Mesh Ewald treatment (Darden et al., 1993) for the electrostatic interactions. For the van der Waals interactions, a switching function was applied between 10 and 12 Å from the solute. The integration time step was 2 fs and coordinates were saved every 1000 steps (2 ps). The duration of the simulations was 125 ns for both the cryo-EM structure and the model of the open form. For both simulations, RMSDs were calculated on all Cα atoms except those belonging to the flexible domains [Lasso (residues 1–65), Regulatory Insertion (residues 404–435), ECL4 (residues 886–909), and Linker Insertion [residues 1169–1200)]. RMSDs reach a plateau after ∼25 ns with averaged values (on the last 100 ns) of 1.8 ± 0.2 and 2.3 ± 0.2 Å, respectively, for the cryo-EM structure and the model of the open MD simulations. Contacts between W361 and other amino-acids or lipids were obtained using VLDM which is based on a Laguerre representation of macromolecules (Esque et al., 2010, 2011). 3D structure representations were made using Pymol (The PyMOL Molecular Graphics System, Version 3.0 Schrödinger, LCC) or Chimera (Pettersen et al., 2004).

### Statistics

Results are expressed as the means ± Standard Error of the Mean (SEM) of *n* observations. All statistical tests were performed using GraphPad Prism version 5.0 (GraphPad Software, San Diego, CA, United States). Datasets were compared using Student’s t-tests. Differences were considered statistically significant at *p* < 0.05. ns, no significant difference; ^∗^*p* < 0.05; ^∗∗^*p* < 0.01; and ^∗∗∗^*p* < 0.001.

## Results

### Expression and Maturation of W361R-CFTR

**FIGURE 1:**
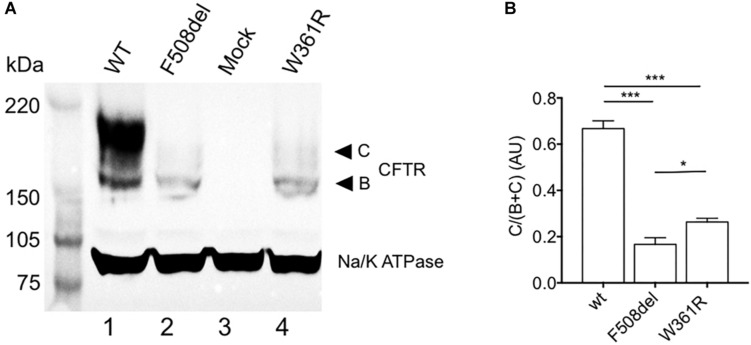
Maturation profile of the W361R mutant. Maturation analysis of WT and mutant CFTR channels. **(A)** Whole cell lysates from HEK cells expressing WT or mutant CFTR proteins analyzed by SDS-PAGE and western blotting with an anti-CFTR antibody. Arrows on the right show the core-glycosylated EGFP-CFTR band B and the mature-glycosylated EGFP-CFTR band C. The housekeeping Na/K ATPase protein (100 kDa) was used as a loading control. **(B)** Bar chart showing processing rate of WT and mutant CFTR proteins (AU: Arbitrary Unit) from different cell lysates and independent gels (*n* = 4). ns, non-significant; *p < 0.05; ***p < 0.001. Error bars, SEM.

To study the maturation of W361R-CFTR, we first performed western blot experiments from HEK293 cells transiently transfected with either WT or mutant forms of CFTR. As expected, the WT-CFTR biochemical profile showed the core glycosylated, B-band, and a fully glycosylated, C-band, on western blots (Figure 1A, lane 1), while the well-known class-2 maturation mutant, F508del-CFTR, showed only the core glycosylated B-band (Figure 1A, lane 2). Interestingly, W361R-CFTR showed a very weak mature C-band (Figure 1A, lane 4). This difference in maturation between the two class-2 mutations was confirmed by densitometry analysis (Figure 1B). A similar biochemical profile was observed when W361R-CFTR proteins were expressed in different cell models (BHK and Hela cells, data not shown). Mock transfected cells (HEK293 cells expressing pEGFP-C1 vector without the CFTR coding sequence) were used as negative control (Figure 1A, lane 3). No band was revealed by the anti-CFTR antibody, confirming the specificity of the antibody.

### Function of W361R Mutant-CFTR

**FIGURE 2:**
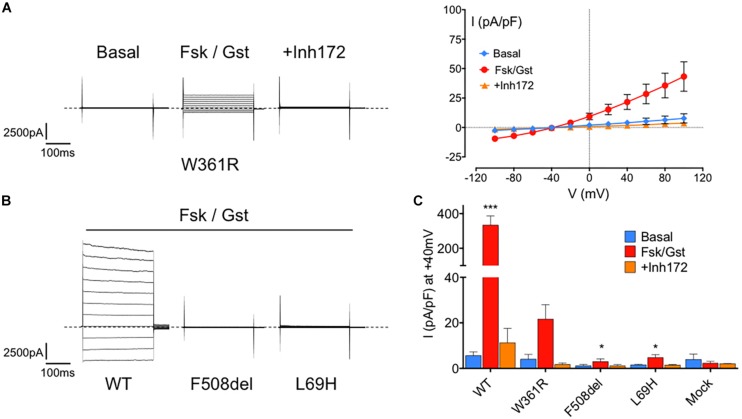
Functional assessment of the W361R-CFTR by electrophysiological recordings. **(A)** Whole cell chloride current of HEK293 cells expressing W361R-CFTR obtained in basal condition, in the presence of 10 μM Fsk + 30 μM Gst or 10 μM CFTR_Inh_172. Representative current traces of mutants (left panel) and corresponding current–voltage relationships normalized by cell capacitance (right panel). Error bars, SEM, *n* = 6. Dotted lines represent the zero current level. **(B)** Whole cell chloride current of HEK293 cells expressing WT-CFTR, F508del-CFTR and L69H-CFTR obtained in the presence of 10 μM Fsk + 30 μM Gst. Dotted lines represent the zero current level. **(C)** Bar chart comparing whole cell current densities obtained in basal condition, in the presence of 10 μM Fsk + 30 μM Gst or 10 μM CFTR_Inh_172 for WT and all mutated CFTR studied. Mock cells expressing empty EGFP-C1 plasmid were added. Statistics compare means of current density of each CFTR tested to W361R in Fsk/Gst condition: *p < 0.05; ***p < 0.001. Error bars, SEM, *n* = 3–7.

We then studied the Cl^–^ channel function of W361R-CFTR by whole cell, patch clamp experiments. Figure 2A shows representative whole cell recordings taken from HEK293 cells expressing W361R-CFTR under basal conditions, and in the presence of 10 μM Fsk + 30 μM Gst or 10 μM of CFTR_*inh*_172 (left panel) and the corresponding current density/voltage (I/V) relationships (right panel). In the presence of Fsk/Gst, the W361R-CFTR mutant exhibited a significant time-independent chloride current completely abolished by CFTR_*inh*_172. For comparison, Figures 2B,C show whole cell Cl^–^ currents recorded for WT-CFTR and two CFTR class-2 mutants, F508del-CFTR and L69H-CFTR. Figure 2B shows the CFTR current traces obtained in the presence of the CFTR activation cocktail (Fsk 10 μM + Gst 30 μM) for the three different CFTR variants. Figure 2C summarizes the current densities recorded under basal conditions, in the presence of Fsk/Gst, and in the presence of CFTR_inh_172. Although the response to Fsk/Gst cocktail for W361R-CFTR was significantly decreased compared to WT-CFTR (current density at +40 mV: 21.65 ± 6.3 pA/pF, *n* = 6 and 333.5 ± 53 pA/pF, *n* = 7, respectively), the W361R-CFTR response was clearly greater than either F508del-CFTR or L69H-CFTR (current density at + 40 mV: 6.33 ± 4.4 pA/pF, *n* = 4 and 4.71 ± 1.4 pA/pF, *n* = 6, respectively). For mock transfected cells, Fsk/Gst had no effect, confirming all currents recorded were carried by CFTR channels.

**FIGURE 3:**
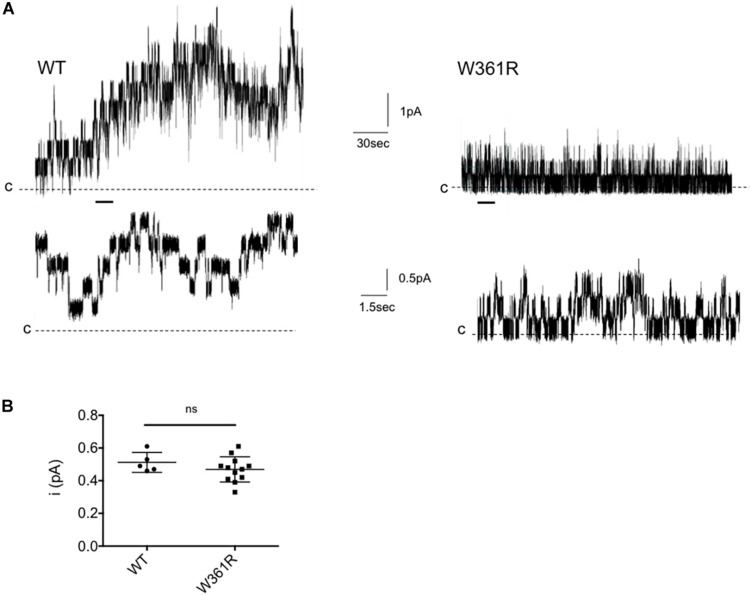
Single channel recordings of W361R. **(A)** Representative recordings of WT and W361R CFTR Cl^–^ channels in excised inside-out membrane patches from HEK cells. The recordings were acquired at room temperature in the presence of 1 mM Mg-ATP and 75 nM PKA in the intracellular solution. Upper traces: 4 min recording; Lower traces: Zoom of the 15 s portions indicated by the bars. Pipette voltage was + 50 mV. Closing states are symbolized by the dashed black lines and upward deflections represent channel openings. **(B)** Single channel conductance of WT and W361R CFTR recorded at V_*m*_ = –50 mV in the presence of 1 mM MgATP + 75 nM PKA. Statistics compare means of wt and W361R single channel conductance. ns, no significant difference. Error bars, SEM, *n* = 5–12.

To investigate further the functional properties of W361R-CFTR, we performed single channel patch clamp experiments, using excised, inside-out patches from transfected HEK293 cells, exposed to 1 mM Mg-ATP and 75 nM PKA to activate CFTR. Figure 3A shows representative traces of microscopic currents for WT-CFTR (left panel) and W361R-CFTR (right panel). The upper traces represent 4 min of recording whereas the lower traces show an expanded 15 s portion of the 4 min trace, as indicated by the bars. We recorded functional W361R-CFTR at the plasma membrane (Figure 3A) in 75% of patches, while WT-CFTR was recorded in 100% of patches (*n* = 6) and F508del-CFTR or L69H were never detected in the absence of correction (*n* = 8). We recorded 1 to 4 channels in excised patches obtained from W361R-CFTR transfected cells, and more than 10 channels for each patch for WT-CFTR, confirming the lower channel density of the W361 mutant. The unitary current (i) of W361R-CFTR was 0.47 ± 0.02 pA at –50 mV which was not significantly different from WT CFTR (i = 0.52 ± 0.03 pA at –50 mV) (Figure 3B). As we did not obtain patches with a single channel for any conditions, we could not investigate the kinetics of the CFTR variants, but these results clearly demonstrate a significant functionality of the W361R-CFTR channels.

### Sensitivity of W361R Mutant to CFTR Modulators

**FIGURE 4:**
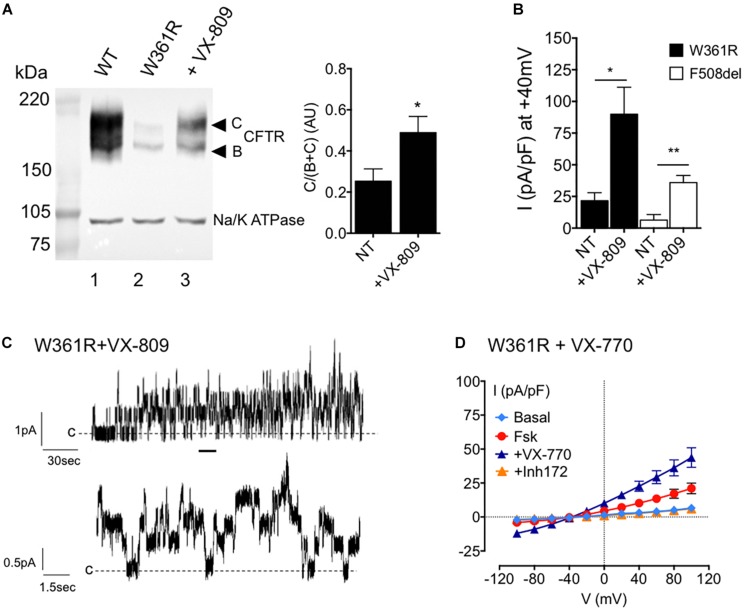
Effects of small molecules on W361R maturation and function. **(A)** Left: Western blot analysis of whole cell lysates from HEK cells expressing WT (lane 1) or W361R without treatment (lane 2) and after a 24 h incubation with 10 μM VX-809 (lane 3) at 37°C. Bands B and C are indicated on the left. The housekeeping Na/K ATPase protein (100kDa) was used as a loading control. Right: Bar chart showing processing rate of W361R-CFTR proteins in non-treated condition (NT) or after corrector incubation with 10 μM VX-809 for 24 h at 37°C (AU, arbitrary unit). Means from different cell lysates and independent gels (*n* = 5). *p < 0.05. Error bars, SEM. **(B)** Whole cell chloride current of HEK293 cells expressing W361R-CFTR or F508del-CFTR not corrected (NT) or treated 24 h with 10 μM VX-809 at 37°C. Current densities obtained in the presence of 10 μM Fsk + 30 μM Gst. *p < 0.05, **p < 0.01. Error bars, SEM, *n* = 6–7. **(C)** Representative recordings of W361R CFTR Cl^–^ channels in excised inside-out membrane patches from HEK cells treated 24 h with 10 μM VX-809 at 37°C. Upper traces: 4 min recording; Lower traces: Zoom of the 15 s portions indicated by the bars. **(D)** Whole cell chloride current of HEK293 cells expressing non-corrected W361R-CFTR. Current–voltage relationships normalized by cell capacitance obtained in basal condition, in the presence of 10 μM Fsk, 10 μM Fsk + 1 μM VX-770 and 10 μM CFTR_Inh_172. Error bars, SEM, *n* = 5.

Because the maturation process of CFTR can be strongly affected by class-2 CF mutations, we studied the effect of the FDA-approved corrector VX-809 (Hudock and Clancy, 2017) on the W361R-CFTR variant, by western blot and patch clamp (Figure 4). First, we detected an increase of the mature C-band of W361R-CFTR in western blot experiments when cells were incubated for 24 h at 37°C with 10 μM VX-809 (Figure 4A, lane 3). Densitometry analyses confirmed a significant, twofold, correction of the maturation defect.

Then whole cell patch clamp current recordings from HEK293 cells expressing corrected W361R-CFTR and corrected F508del-CFTR confirmed the western blot results (Figure 4B). Figure 4B shows the current densities obtained in the presence of Fsk/Gst under non-treated (NT) conditions, or after 24 h incubation with 10 μM VX-809 at 37°C (+ VX-809). In the presence of VX-809, the activator cocktail induced a larger CFTR current for both mutants. Moreover, corrected W361R-CFTR showed a better functionality than the corrected F508del-CFTR (at +40 mV: 89.9 ± 21.4 pA/pF *n* = 7 and 35.9 ± 5.6 pA/pF *n* = 6, respectively). Inside-out recordings confirmed there was an increase in W361R channels present at the plasma membrane. We recorded channel activity in 100% of patches from corrected W361R-CFTR transfected cells (*n* = 6) with an estimated number of channel between 4 and 10. Unitary conductance was not modified after the VX-809 treatment (i = 0.51 ± 0.02 pA at –50 mV).

Finally, we tested the effect of the FDA-approved CFTR modulator VX-770 on non-corrected W361R-CFTR by whole cell patch clamp. Figure 4D shows the I/V relationships under basal conditions and after sequential addition of 10 μM Fsk, 1 μM VX-770, and 10 μM CFTR_inh_172. VX-770 clearly potentiated the Fsk-activated current and caused a further twofold increase in whole cell current (at +40 mV: 10.2 ± 2 pA/pF for Fsk alone and 22.6 ± 3.7 for Fsk + VX-770, *n* = 5). This VX-770 dependent current was completely abolished by CFTR_inh_172. These results clearly show that small molecules correct both the maturation and the function of the W361R-CFTR mutant.

### Position of W361 on the CFTR 3D Structures and Possible Involvement in Protein-Lipid Interactions

**FIGURE 5:**
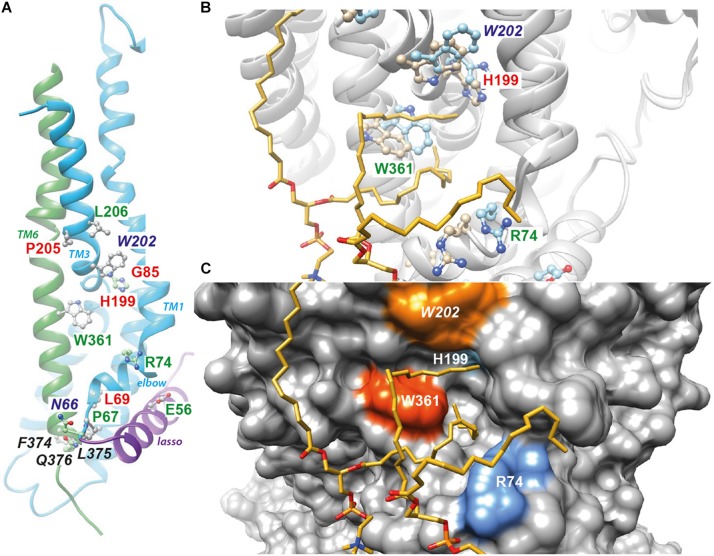
Position of W361 on the CFTR 3D structure. **(A)** Position of W361 on the experimental 3D structure of the ATP-bound, phosphorylated 3D structure of human CFTR [cryo-EM 3D structure, pdb 6MSM (Zhang et al., 2018)]. The end of the lasso structure is shown in purple. The N- (N66) and C-ter (Q376) extremities of CFTR MSD1 are in contact. The amino acids not reported in italics are affected by disease-associated mutations. Amino acids in green and red correspond to mild and severe class-2 mutations, respectively, as discussed in the text. **(B)** The same 3D structure and amino acids before (light blue) and after MD simulations in the presence of lipids. The two POPC molecules, whose acyl chains filled the groove formed by the elbow helix, TM1, TM3 and TM6, are shown. **(C)** Surface representation of the CFTR 3D structure, highlighting the groove. Supplementary Figure 1 reports the same data for the model of the CFTR open channel (Hoffmann et al., 2018), while Supplementary Figure 2 reports on the contacts established by W361 with other amino acids and POPC molecules along the MD simulations. Supplementary Figure 3 illustrates the common presence of the groove filled by lipids in two different simulations performed in similar conditions using cryo-EM 3D structures (3D representation and contact maps).

Cryo-electron microscopy (cryo-EM) of the full-length CFTR protein has given information about both an ATP-free, non-phosphorylated apo conformation [human and zebrafish CFTR (Zhang and Chen, 2016; Liu et al., 2017)] and an ATP-bound, phosphorylated, yet closed conformation [human and zebrafish CFTR (Zhang et al., 2017, 2018)]. These ATP-free and ATP-bound conformations, both corresponding to closed channels, are very similar in the W361 area (root mean square standard deviation (RMSD) of 2.95 Å for 204 superimposed Cα). In the close 3D neighborhood of W361 are found E56, P67, R74, G85, H199, P205 and L206, amino acids that are affected by CF-causing mutations [(Veit et al., 2016) and CFTR2 database (see text footnote 1), Figure 5A]. This region, including the N- (elbow and TM1) and C-termini (TM6) of membrane spanning domain 1 (MSD1) as well as the N-terminal extension (in the so-called lasso conformation), thus appears sensitive to deleterious mutations. It is worth noting that this region is also found in a model of the open form of the CFTR channel (Supplementary Figure 1A, RMSD 4.7 Å on 204 Cα, Mornon et al., 2015). This model, supported by experimental data (El Hiani and Linsdell, 2015) and consistent with the cryo-EM 3D structure at the level of individual blocks of TM helices (Hoffmann et al., 2018), gives insight into an alternative conformation of the CFTR channel, with a different configuration of the TM7-TM8 hairpin as well as of the N-terminal extension (lasso in the cryo-EM 3D structure), free for interaction with cytosolic partners (Supplementary Figure 1A).

We performed all-atom MD simulations of wild-type CFTR embedded in a lipid environment, starting with both the experimental (closed channel) and theoretical (open channel) 3D structures. Cryo-EM 3D structures were very similar before and after MD simulations (Figure 5B), with a rotation of the W361 side chain, while lipid (POPC) acyl chains filled the groove formed by TM1, TM3 and TM6, as well as the elbow helix, establishing stable contacts in particular with aromatic (W361, W202) and basic residues (R74, R202) (Figures 5B,C and Supplementary Figure 2A). These global features were also observed in another MD simulation performed in similar conditions using cryo-EM 3D structures, irrespective of the natural variability of side chain positions between the simulations or within the same simulation (Supplementary Figure 3). A similar situation is observed after MD simulation of the WT CFTR model of the open channel (Supplementary Figures 1B,C, 2B).

From these data, we suggest that the introduction of an arginine residue instead of tryptophan at position 361, near to the beginning (N66 in transmembrane helix TM1) and the end (Q376 in TM6) of MSD1, which are in close proximity to a groove likely occupied by lipids, may disturb the proper folding and stability of the mutated protein. This suggestion is consistent with the biochemical and electrophysiological profiles we have obtained experimentally. This hypothesis is supported by the fact that F374 and L375, at the end of TM6, also play critical role for MSD1 folding (Ren et al., 2013).

## Discussion

In the present work, we have investigated the effects of the rare CFTR mutation W361R. We found a strong decrease in the levels of the fully glycosylated mature form compared to WT-CFTR, a hallmark of class-2 CF mutations (Kerem, 2006). Without any correction, whole cell patch clamp recordings detected a significant chloride current following cAMP stimulation, which was completely abolished by the specific CFTR inhibitor, CFTR_Inh_172. Although the magnitude of the W361R-CFTR current was much less than WT-CFTR, it was significantly greater than either F508del-CFTR or L69H-CFTR, two severe class-2 mutations. Inside-out patch clamp experiments confirmed the presence of functional W361R-CFTR at the plasma membrane with a normal conductance but a low channel density. W361R-CFTR maturation could be corrected by the potent CFTR corrector VX-809 (Hudock and Clancy, 2017). Moreover, after VX-809 treatment, we recorded in our inside-out patches a higher current density for the mutant W361R-CFTR compared to F508del-CFTR. This is also consistent with an increase in channel density. We also showed that the W361R mutation did not prevent the potentiation of the Cl^–^ current by VX-770, suggesting that a CF patient bearing this CFTR variant would respond to VX-770 treatment.

W361 is located in the TM6 helix, within a region bringing together the MSD1 elbow helix, TM1, TM3 and TM6, and forming a groove at the protein/membrane interface that can be stably occupied by lipid acyl chains (Figure 5). This region appears vulnerable to mutations, as several CF class-2 mutations are found there, forming a mutation hotspot, which are associated with both mild and severe maturation defects (Ren et al., 2013) and CFTR2 database (see text footnote 1). Hence, E56K, P67L, R74W, L206W and now W361R, all cause a decrease in the mature form of CFTR, but still show a significant chloride conductance without any treatment (Clain et al., 2005; Van Goor et al., 2014). In contrast, L69H, G85E or H199Y present a profile similar to F508del with the absence of mature form of CFTR and no channel expression in the absence of correction (Van Goor et al., 2014; Sharma et al., 2015). The severity of the L69, G85 and H199 mutations could be explained by the important role these amino acids play in the stability of the 3D structure, as they are embedded in the MSD1 core and/or involved in inter-helix packing. In contrast, W361, E56, P67, R74, or L206, seem to play a less essential role by being more exposed to the solvent, interacting with lipids and/or present in more flexible regions (Callebaut et al., 2017a; Liu et al., 2017). More particularly, W361 is located at the membrane-water interface, a position widely observed for tryptophan residues in membrane proteins (Yau et al., 1998), which is correlated to its versatile molecular properties [largest non-polar surface area and polarizability (H-bonds, cation-π or CH-π contacts)]. Given its role in specific protein-lipid interactions, tryptophan has been shown to be a key actor in membrane protein folding and stabilization (Sanchez et al., 2011). By showing specific interactions with lipids, our MD simulations suggest that CFTR W361 might also play an important role in MSD1 conformation.

VX-809 has been shown to partially correct F508del-CFTR folding by positively affecting the MSD1 conformation, thereby improving the efficiency of interactions with other domains (Ren et al., 2013). A VX-809-binding site has been proposed in the vicinity of the MSD1 class-2 mutation hotspot reported here (Laselva et al., 2018; Molinski et al., 2018). Binding to this site, or sites located nearby, might improve the stability of this fragile region which is, like the ICL4:NBD1 interface, more sensitive to any change than other parts of the protein (Zhang and Chen, 2016; Callebaut et al., 2017b). This hypothesis requires to be further explored, especially as other possible VX-809-binding sites have been proposed (Okiyoneda et al., 2013; Hudson et al., 2017).

To conclude, these results on W361R support the revision of the mutation classification that we (Froux et al., 2018) and others proposed (Veit et al., 2016) with a division of class-2 mutations into two sub-categories, which either cause a severe or a mild maturation defect, and which induce a more or less severe CF phenotype. W361R would be classified as a mild subclass-2 CF mutation having residual chloride channel activity, whereas F508del and L69H are severe subclass-2 CF mutations with no chloride channel activity. This mild defect could explain the late CF diagnosis for patients carrying the W361R mutation (Bienvenu et al., 1994), as well as for other mutations in the neighborhood of W361, such as P67L (Sabusap et al., 2016) or in different regions such as V232D (Therien et al., 2001), G622D (Vankeerberghen et al., 1998; Billet et al., 2010) or R1070W (Krasnov et al., 2008), collectively belonging to this mild subclass-2 category.

## Data Availability Statement

All datasets generated for this study are included in the article/Supplementary Material.

## Author Contributions

AB, AE, MJ-S, BH, J-PM, IC, and FB designed the experiments. AB and MJ-S performed and analyzed the biochemical experiments. AB performed and analyzed the patch clamp experiments. AE performed the molecular dynamics simulations. AE, BH, J-PM, and IC analyzed the structural data. AB, AE, IC, and FB wrote the manuscript.

## Conflict of Interest

The authors declare that the research was conducted in the absence of any commercial or financial relationships that could be construed as a potential conflict of interest.

## Footnotes

1 https://www.cftr2.org/

## Abbreviations

CF: cystic fibrosis
CFTR: cystic fibrosis transmembrane conductance regulator
GFP: green fluorescent protein
WT-CFTR: wild-type CFTR.
