# Supplementary material for: Functional and Pharmacological Characterization of the Rare CFTR Mutation W361R
Source: Front Pharmacol. 2020 Mar 17;11:295. doi: 10.3389/fphar.2020.00295 (PMC7092619; doi:10.3389/fphar.2020.00295)
Supplement: Supplementary file 1 [file Presentation_1.pptx]

## Slide 1
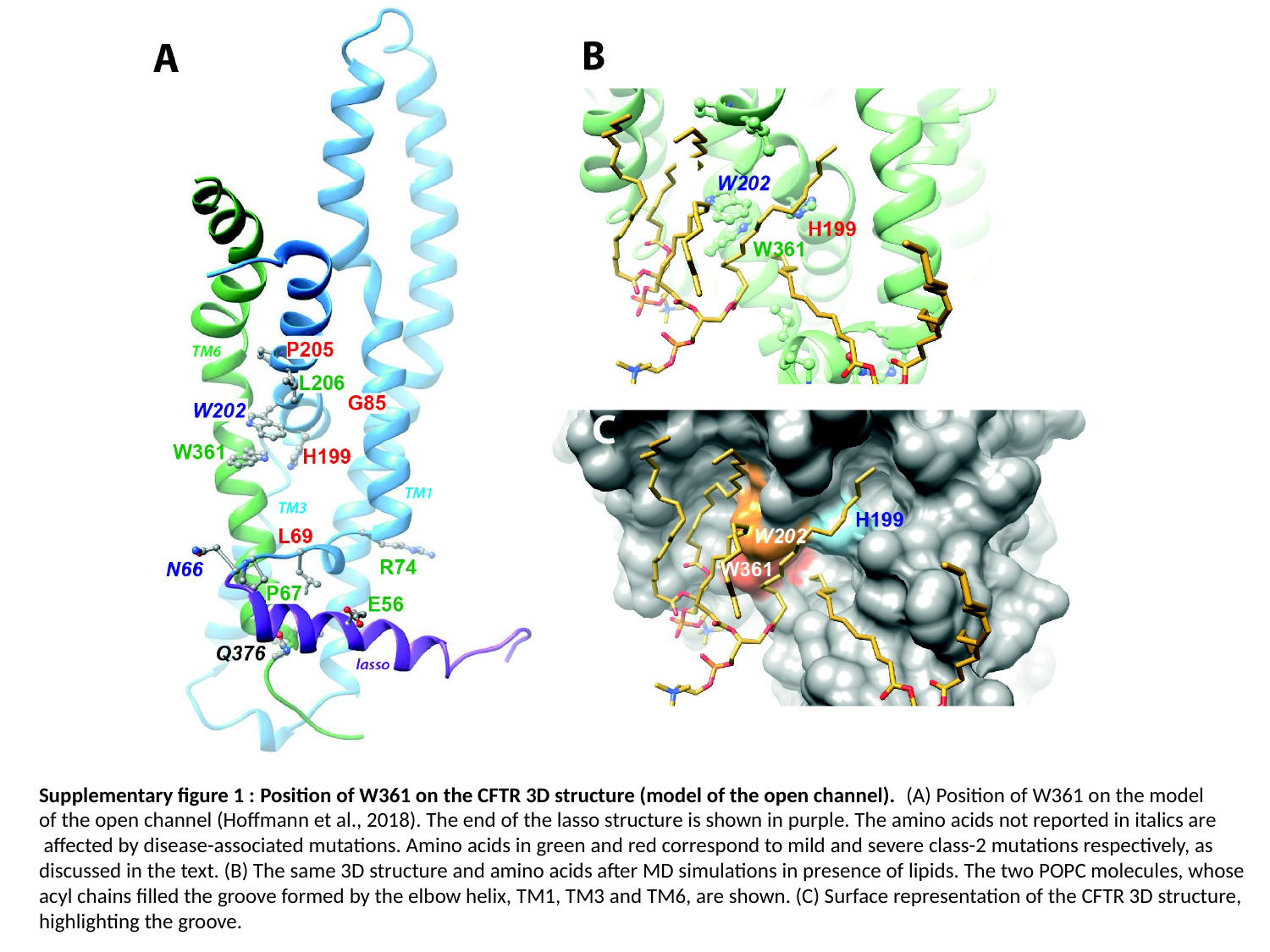

Supplementary figure 1 : Position of W361 on the CFTR 3D structure (model of the open channel). (A) Position of W361 on the model
of the open channel (Hoffmann et al., 2018). The end of the lasso structure is shown in purple. The amino acids not reported in italics are
 affected by disease-associated mutations. Amino acids in green and red correspond to mild and severe class-2 mutations respectively, as
discussed in the text. (B) The same 3D structure and amino acids after MD simulations in presence of lipids. The two POPC molecules, whose
acyl chains filled the groove formed by the elbow helix, TM1, TM3 and TM6, are shown. (C) Surface representation of the CFTR 3D structure,
highlighting the groove.

## Slide 2
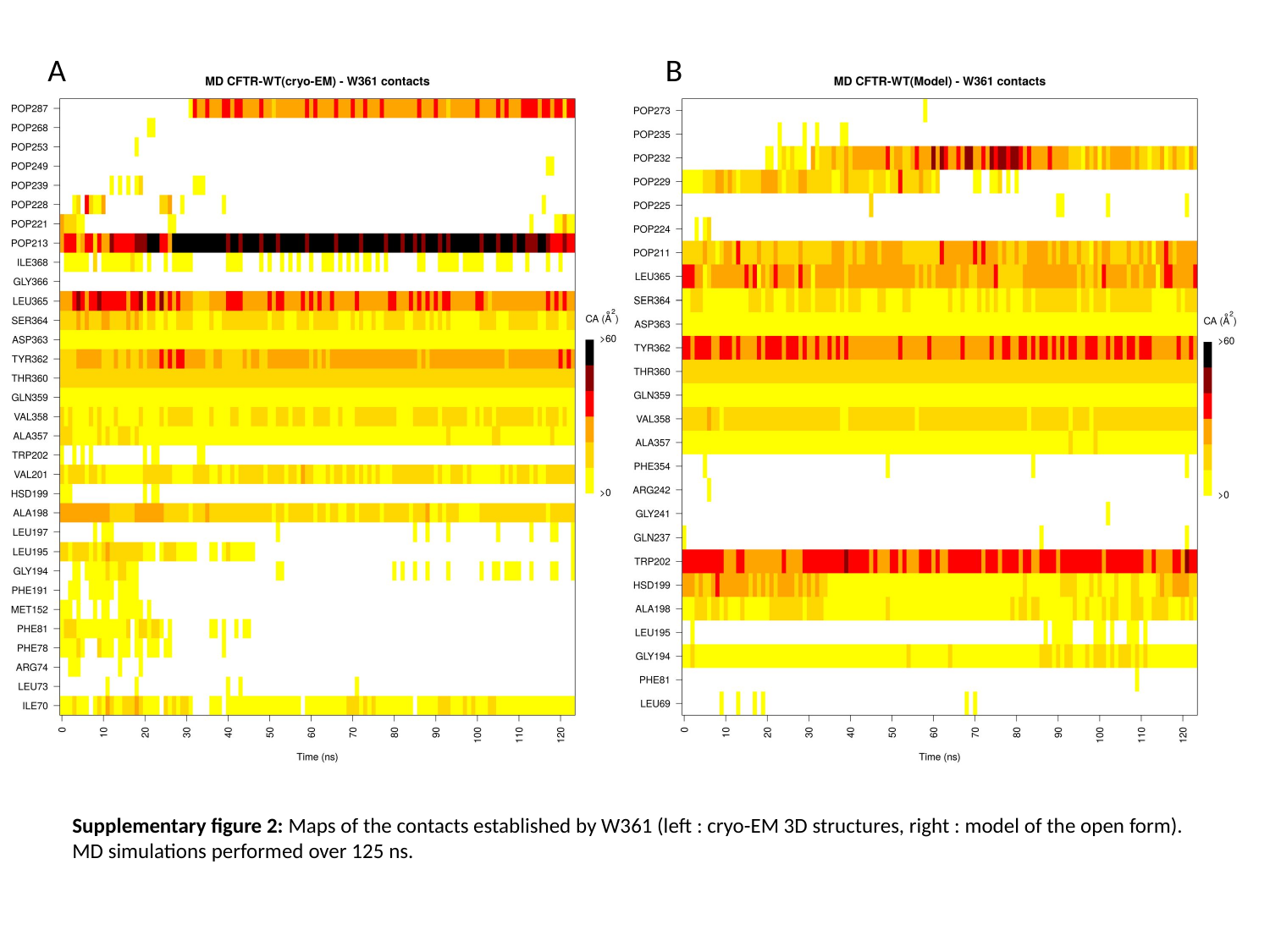

A
B
Supplementary figure 2: Maps of the contacts established by W361 (left : cryo-EM 3D structures, right : model of the open form).
MD simulations performed over 125 ns.

## Slide 3
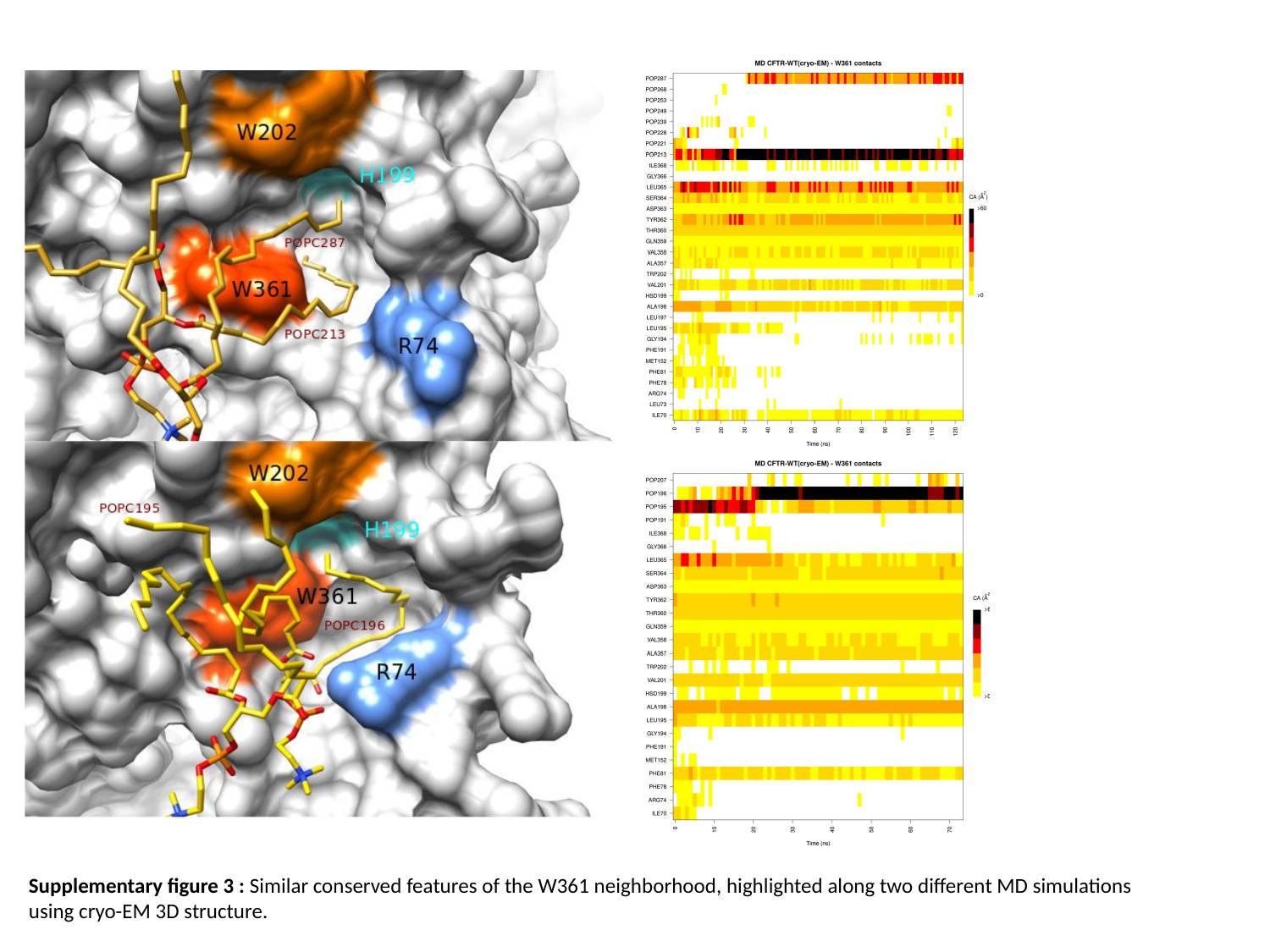

Supplementary figure 3 : Similar conserved features of the W361 neighborhood, highlighted along two different MD simulations
using cryo-EM 3D structure.
